# Signatures of balancing selection are maintained at disease resistance loci following mating system evolution and a population bottleneck in the genus Capsella

**DOI:** 10.1186/1471-2148-12-152

**Published:** 2012-08-21

**Authors:** Gesseca Gos, Tanja Slotte, Stephen I Wright

**Affiliations:** 1 Department of Biology, York University, 4700 Keele Street, Toronto, ON M3J 1P3, Canada; 2 Department of Evolutionary Biology, EBC, Uppsala University, Norbyägen 18D, Uppsala 75236, Sweden; 3 Department of Ecology and Evolutionary Biology University of Toronto, 25 Willcocks Street, Toronto, ON M5S 3B2, Canada

**Keywords:** Capsella *grandiflora*, Capsella rubella, R-genes, Balancing selection, Relaxed constraint, Population bottleneck

## Abstract

**Background:**

Population bottlenecks can lead to a loss of variation at disease resistance loci, which could have important consequences for the ability of populations to adapt to pathogen pressure. Alternatively, current or past balancing selection could maintain high diversity, creating a strong heterogeneity in the retention of polymorphism across the genome of bottlenecked populations. We sequenced part of the LRR region of 9 NBS-LRR disease resistance genes in the outcrossing *Capsella grandiflora* and the recently derived, bottlenecked selfing species *Capsella rubella*, and compared levels and patterns of nucleotide diversity and divergence with genome-wide reference loci.

**Results:**

In strong contrast with reference loci, average diversity at resistance loci was comparable between *C. rubella* and *C. grandiflora*, primarily due to two loci with highly elevated diversity indicative of past or present balancing selection. Average between-species differentiation was also reduced at the set of R-genes compared with reference loci, which is consistent with the maintenance of ancestral polymorphism.

**Conclusions:**

Historical or ongoing balancing selection on plant disease resistance genes is a likely contributor to the retention of ancestral polymorphism in some regions of the bottlenecked *Capella rubella* genome.

## Background

The prevalence of adaptive evolution in natural populations is one of the most widely investigated questions in evolutionary genetics. The long-held theory that the vast majority of mutations are either neutral or strongly deleterious [1], has recently come into question, in light of evidence to the contrary. Several model organisms, including *Drosophila melangaster*[2]*, Mus musculus*[3], *Escherichia coli*[4], *Capsella grandiflora*[5], and several Helianthus species [6], are estimated to have large proportions (40-50%) of amino acid divergence driven to fixation by positive selection. Estimates for other organisms, using comparable approaches, are much lower and thus more consistent with the neutral theory. These include humans [7,8], and *Arabidopsis thaliana*[5,9-11]. The presence and prevalence of species-wide fixations of beneficial mutations across genomes therefore appears to vary among taxa, and is currently a focal point of interest in the field of molecular evolution.

One possible reason for differences in the amount of adaptive evolution between species is a difference in effective population size [1,12]. Effective population size influences the substitution rate of beneficial mutations. Smaller populations will have lower rates of adaptive substitution compared to larger ones, in addition to having an increased number of slightly deleterious mutations fixed by genetic drift [13]. This will reduce the efficiency and prevalence of both positive and purifying selection in the genome. The genetic model plant *Arabidopsis thaliana* has been shown to have less efficient positive and purifying selection compared to its close relative *Capsella grandiflora*, a result that is consistent with the higher population structure, recent range expansion, and lower effective population size of *A. thaliana*[5]. A comparison of another genetic model system, the house mouse (*Mus musculus*), to humans showed a similar pattern according to their differences in effective population size [3], and an analysis of six sunflower species (Helianthus) showed a positive correlation between effective population size and the rate of adaptive evolution by positive selection [6].

In addition to the effects on the rates of positive and negative selection, species’ differences in effective population sizes can influence the strength and impact of balancing selection. On the one hand, severe reductions in effective population size could lead to a loss of the diversity that is maintained by balancing selection, which could have important deleterious consequences. For instance, when considering a species’ ability to maintain resistance to parasites, balancing selection is critical at the major histocompatibility locus (MHC) in vertebrates (fish [14]; prairie chickens [15]; honeycreepers [16]; voles [17]; deer mice [18], foxes [19]), and balancing selection has also been shown in a number of cases in plants at disease resistance (R) loci (Arabidopsis [20-22]; tomato [23]; rice [24]; grasses [25]. Strong reductions in effective size could greatly reduce the possibilities of maintaining resistance in populations [19]. Alternatively, strong balancing selection, either past or present, could maintain high polymorphism in heavily bottlenecked populations at specific loci, showing a pronounced retention of diversity at specific regions of the genome, despite genome-wide loss of diversity. At the MHC locus, several cases of striking retention of diversity under severe bottlenecks have been found [16,19]. In other cases, a loss of balancing selection has been observed at MHC [15]. A similar pattern has been observed at the plant self-incompatibility (SI) locus, as long-term allelic variation maintained by balancing selection at SI was lost following an ancient population bottleneck of the Solanaceae [26].

Here, we investigate the comparative population genetics of a set of disease resistance (R) genes in two plant species, *Capsella grandiflora* and *Capsella rubella*, two members of the Brassicaceae. *Capsella grandiflora* is an annual, self-incompatible herb that is closely related to the genetic model *Arabidopsis thaliana* (~20 MYA divergence time, [27]). *Capsella rubella*, a recently diverged relative, is self-fertilizing, and has experienced a severe population bottleneck. The bottleneck and change in mating system resulted in a major reduction in genetic diversity and effective population size [28]. The fact that speciation is recent, coupled with the severity of the diversity reduction, make these two species a useful system in which to explore the evolutionary fate of selected genes, in light of a dramatic shift in genetic background.

*Capsella grandiflora* is native to Western Greece, and its geographic range is largely restricted to this area, in addition to small populations in Albania and Northern Italy [29,30]. Its effective population size is large, approximately 500,000 individuals, and appears to have been relatively stable over a long time period, as it shows no evidence for recent changes in population size [28]. There is also relatively little population structure in this species, and the effective rate of recombination is high [28,31]. As stated earlier, selection has been inferred to be highly efficient in this species, with over 40% of amino acid divergence inferred to be subject to positive selection [5].

*Capsella rubella* diverged from *C. grandiflora* in a single event that is estimated to have taken place within the last 20,000 years [28,32]. Speciation was associated with the breakdown of self-incompatibility in *C. rubella*, and this species has evolved to be highly self-fertilizing [33]. The transition in mating system was followed by a geographic range expansion throughout most of Southern Europe, as well as Middle Europe, North Africa, Australia, and North and South America [29,30]. Genetic diversity is greatly reduced in *C. rubella* compared to *C. grandiflora*, even more so than would be expected from inbreeding alone, due to a nearly complete population bottleneck [28]. *Capsella rubella* therefore has a much smaller effective population size than *C. grandiflora*, approximately 100 to 1500 fold smaller, as well as a lower effective recombination rate [28]. These two species represent a recent and rapid dramatic shift in genomic characteristics, including a mating system transition, a reduction in genetic diversity and effective population size following a population bottleneck, and recent widespread expansion in geographic range. Despite this severe bottleneck, however, there is strong heterogeneity in the retention of polymorphism at different loci in *C. rubella*[28,31]. One possible explanation for this heterogeneity could be the maintenance of balancing selection and/or historical balancing selection having led to a higher retention of diversity at a subset of genomic regions.

The genes we investigated here are a subset of the genes thought to be involved in plant immune system function, the disease resistance (R) genes. These genes are abundant in every plant species investigated to date [34], and can be subdivided into classes based on their coding domains. The largest class are characterized by a nucleotide binding site combined with a region of leucine-rich repeats, referred to as the NBS-LRR region, which is thought to be the site of pathogen recognition. The R-genes are typically characterized by a gene-for-gene interaction [35,36], whereby each gene in the plant specifically recognizes an avirulence (avr) gene in the pathogen, and recognition triggers a defense response in the plant.

Evidence for natural selection on plant R-genes, including positive and balancing selection, has been well documented for several well-characterized genes in the genetic model *Arabidopsis thaliana*, including RPM1, RPS2, RPS4, RPS5, RPP1, RPP13, and RPP8 [20,22,37-41]. The majority of clear evidence for selection was found to be balancing selection in these genes, with the exception of RPS4, which has undergone a recent selective sweep [40]. Evidence for balancing selection has also been found in *Arabidopsis lyrata*[42,43], and differences in the types of selection at the same R-genes have been observed between *Arabidopsis thaliana* and *A. lyrata.* For example, *A. lyrata* contains a segregating presence-absence polymorphism at the R-gene RFL1 that is not observed in *A. thaliana* (RPS5) [42]. Polymorphism patterns at the R-gene RPP13 indicate positive selection at *A. thaliana,* but show purifying selection in *A. lyrata*[43]. Plant R-genes often segregate for alleles that confer either resistance or susceptibility to a specific pathogen. In the case of balancing selection, both alleles are maintained over long time periods. The mechanism for this is proposed to be frequency-dependent selection, where resistance genes are advantageous when the pathogen is common, but incur a fitness cost when pathogens are rare. The result is a cycle of resistance and susceptibility alleles that alternate in frequency following the dynamics of the pathogen population [20,22]. Although it has not been demonstrated directly to date, another possible mode of balancing selection could arise when alleles at a single locus show varying specificities to different pathogen strains, and they are subject to frequency-dependent selection [23]. On the other hand, R-genes may also often experience relaxed constraint under conditions where target pathogens are absent and there is no cost of resistance [44]. In general, large surveys of R-genes generally show more clear evidence for balancing selection than positive selection, although this is still only at a subset of loci [40,45]. Overall, the patterns in Arabidopsis species indicate that new R-gene alleles are constantly being generated, but only briefly maintained, which is a scenario closer to diversifying selection [42,45].

Here, we aim to investigate the consequences of a severe population bottleneck and mating system transition on the polymorphism patterns at R-genes in the two Capsella species. Genetic signatures of natural selection that are present in *Capsella grandiflora* at disease resistance genes may be diminished or absent in *C. rubella* if the bottleneck has effectively eroded allelic variation generated by selection. However, if the selective signatures at R-genes in *C. grandiflora* are also present in *C. rubella*, this would suggest that strong balancing or diversifying selection associated with pathogen resistance, or a history of such selection in *C. grandiflora*, has caused the allelic diversity in these regions to be maintained in *C. rubella*, despite a genome-wide loss of neutral variation. We take advantage of an extensive dataset on coding region polymorphism in the two species at 283 reference genes, in order to contrast R-gene diversity in a comparable population sample with the genome-wide pattern.

## Results

Diversity levels at R genes in both *C. rubella* and *C. grandiflora* are highly variable across loci (Table 1). Nevertheless, average non-synonymous diversity (π, θ_w_) and divergence from the outgroup *Arabidopsis thaliana* (Ka) are significantly greater than the genome-wide average (p < 0.001) for the R-genes in both species, according to the permutation tests. All Ks values, including averages, were not significantly elevated (Table 1). In *C. rubella*, average synonymous diversity at R-genes is also greater than the genome average (p < 0.001), but this is not the case in *C. grandiflora* (Table 1). The summary statistics of many of the individual R-genes fall within the upper 2.5% tail of the genome-wide distribution, as indicated with asterisks (Table 1). The locus AT1G56540 has particularly elevated diversity, both synonymous and non-synonymous, in both species, in addition to showing increased non-synonymous divergence and Tajima's D statistic. Additionally, locus AT1G63730, has extremely high polymorphism levels in *C. rubella* alone, and it is these two loci that are largely driving elevated diversity levels in *C. rubella* R genes (Table 1). Many individual resistance loci have higher levels of non-synonymous divergence than the genome average (p < 0.025) in one or both species, including AT1G17600 (*C. grandiflora*), AT1G63730 (*C. rubella*), AT1G27170, AT1G54540, and AT1G64070 (Table 1).

**Table 1:** Individual and average summary statistics for the disease resistance genes

**Polymorphism Type:**			**Synonymous**							**Non-Synonymous**								
**Species**	**Locus**	**n**^**1**^	**# sites**^**2**^	**θ**_**w**_^**3**^		**π**^**4**^		**Ks**^**5**^	**D**^**6**^	**# sites**^**7**^	**θ**_**w**_^**8**^		**π**^**9**^		**Ka**^**10**^		**D**^**11**^	
*Capsella grandiflora*	At1g12290	16	126	0.0120		0.0071		0.2028	-1.3272	420	0.0007		0.0008		0.0446		0.1557	
	At1g17600	10	123	0.0029		0.0038		0.2747	0.8198	444	0.0016		0.0019		0.0998	*	0.5259	
	At1g27170	14	134	0.0070		0.0059		0.2970	-0.4937	415	0.0038		0.0026		0.1187	*	-1.0908	
	At1g56540	6	69	0.0637	*	0.0776	*	0.3001	1.3080	222	0.0197	*	0.0240	*	0.1219	*	1.3080	*
	At1g63730	16	121	0.0025		0.0019		0.2096	-0.4483	398	0.0053		0.0038		0.0770		-0.9903	
	At1g63740	12	129	0.0258		0.0345		0.2172	1.4014	420	0.0087		0.0076		0.0547		-0.4916	
	At1g64070	8	143	0.0403		0.0438		0.2976	0.4427	472	0.0262	*	0.0310	*	0.1165	*	0.9753	
	At3g50950	16	127	0.0213		0.0255		0.2569	0.7168	428	0.0014		0.0011		0.0124		-0.5778	
	At5g17680	16	129	0.0140		0.0160		0.1530	0.4735	414	0.0029		0.0025		0.0427		-0.4684	
	R-Genes Average		122	0.0157		0.0173		0.2454	0.1981	404	0.0063	***	0.0064	***	0.0708	***	-0.2452	
	Genome Average		126	0.0224		0.0227		0.2832	0.0132	411	0.0021		0.0019		0.0262		-0.3586	
*Capsella rubella*	At1g12290	7	126	0.0032		0.0023		0.2003	-1.0062	420	0.0000		0.0000		0.0441			
	At1g27170	7	134	0.0000		0.0000		0.2881		415	0.0000		0.0000		0.1171	*		
	At1g56540	7	69	0.0534	*	0.0692	*	0.2999	1.5748	222	0.0147	*	0.0193	*	0.1204	*	1.6416	*
	At1g63730	7	122	0.1442	*	0.1683	*	0.2885	0.9604	397	0.0832	*	0.0971	*	0.1186	*	0.9711	
	At1g64070	7	146	0.0000		0.0000		0.3097		469	0.0009		0.0006		0.1153	*	-1.0062	
	At3g50950	7	127	0.0000		0.0000		0.2494		428	0.0000		0.0000		0.0118			
	At5g17680	7	128	0.0159		0.0111		0.1565	-1.4861	415	0.0039		0.0034		0.0462		-0.5976	
	R-Genes Average		122	0.0310	***	0.0358	***	0.2561	0.0107	395	0.0147	***	0.0172	***	0.0819	***	0.2522	
	Genome Average		126	0.0059		0.0061		0.2826	0.0121	412	0.0007		0.0006		0.0262		-0.0558	

* p < 0.025, ** p < 0.01, *** p < 0.001.

^1^ Sequence sample size.

^2^ Total number of synonymous sites in the sequence.

^3^ The nucleotide diversity statistic θ_w_[46], calculated using only synonymous sites.

^4^ The nucleotide diversity statistic π [47,48], calculated using only synonymous sites.

^5^ Nucleotide divergence from the outgroup *Arabidopsis thaliana*, calculated using only synonymous sites.

^6^ The frequency spectrum statistic Tajima's D [49], calculated using only synonymous sites.

^7^ Total number of non-synonymous sites in the sequence.

^8^ The nucleotide diversity statistic θ_w_[46], calculated using only non-synonymous sites.

^9^ The nucleotide diversity statistic π [47,48], calculated using only non-synonymous sites.

^10^ Nucleotide divergence from the outgroup *Arabidopsis thaliana*, calculated using only non-synonymous sites.

^11^ The frequency spectrum statistic Tajima's D [49], calculated using only synonymous sites.

Differentiation between species is lower than the genome-wide distribution (p < 0.025) in four of the seven R-genes that were investigated in both species (Table 2). The disease resistance genes as a group have significantly lower differentiation than the genome average by an even greater extent (p < 0.001), according to the permutation tests (Table 2). The percentage of unique synonymous polymorphisms is also significantly increased in *Capsella rubella* in the R-genes as a group compared to the genome-wide distribution (p < 0.001) (Table 3). The pattern is largely driven by an extremely high percentage of unique synonymous polymorphisms in *C. rubella* at the highly polymorphic AT1G63730. The two loci AT1G56540 and AT5G17680 also have more unique synonymous polymorphisms in *C. rubella* than the genome average, showing a trend in the same direction. Initially, an extremely low Fst value of 0.00 at AT1G63730 (Table 2), indicating no between-species differentiation, appears to conflict with the vastly different patterns of shared and unique polymorphism between the two species at this locus (Table 3). However, since very little polymorphism is unique to *C. grandiflora*, most of it will be also contained within *C. rubella*, rendering the differentiation between the two species low. Even though *C. rubella* harbors so much of its own unique polymorphism, this is not captured by the Fst statistic, which measures differentiation alone.

**Table 2:** Differentiation of individual and average disease resistance genes between species

** Locus**	**Fst**
At1g12290	0.1938*
At1g27170	0.5481
At1g56540	0.0000*
At1g63730	0.0000*
At1g64070	0.5013
At3g50950	0.4056
At5g17680	0.1245*
R-genes	0.1864**
Genome	0.6008

* p < 0.025 , ** p < 0.001.

**Table 3:** Percentages of shared, unique and fixed polymorphisms by category for individual and average disease resistance genes

**R-Gene**	**% unique synonymous,*C. grandiflora***	**% unique synonymous,*C. rubella***	**% shared synonymous**	**% fixed synonymous**	**% unique non-synonymous,*C. grandiflora***	**% unique non-synonymous, *C. rubella***	**% shared non-synonymous**	**% fixed non-synonymous**
At1g12290	1.00	0.00	0.00	0.00	0.00	0.00	0.00	0.00
At1g27170	1.00	0.00	0.00	0.00	1.00	0.00	0.00	0.00
At1g56540	0.11	0.11	0.78*	0.00	0.14	0.14	0.71*	0.00
At1g63730	0.03	0.97*	0.00	0.00	0.07	0.91*	0.02	0.00
At1g64070	0.69	0.00	0.00	0.31*	0.71	0.00	0.05	0.24*
At3g50950	1.00	0.00	0.00	0.00	1.00	0.00	0.00	0.00
At5g17680	0.44	0.33	0.22	0.00	0.43	0.57	0.00	0.00
R-Gene Average	0.61	0.20*	0.14	0.04	0.48	0.23	0.11	0.03
Genome Average	0.74	0.07	0.11	0.05	0.59	0.12	0.05	0.03

* p < 0.05.

**Figure 1:**
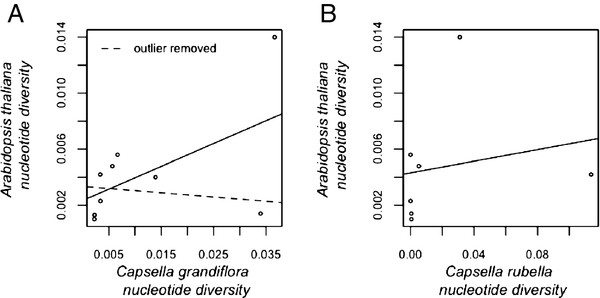
**Correlation in nucleotide diversity between Capsella species and *Arabidopsis thaliana *Correlation between total nucleotide diversity (π) for the R-genes in the two *Capsella *species and total nucleotide diversity (π) in the same set of R-genes in *Arabidopsis thaliana ***[50]**.A**) *Capsella grandiflora* and *Arabidopsis thaliana*. The dashed line represents the correlation between the two species after the outlier, AT1G56540, has been removed. **B**) Correlation between R-genes in *Capsella rubella* and *Arabidopsis thaliana*.

Interestingly, the levels of nucleotide diversity (π) at the R-genes in *Capsella grandiflora* show a strong correlation with levels of nucleotide diversity (π) for the same sample of R-genes in A. thaliana [45] (Figure 1A; p = 0.0175, r² = 0.768). However, when the outlier AT1G56540, is removed, the correlation disappears (Figure 1A; p = 0.748, r² = 0.136), suggesting the pattern may be driven primarily by a shared selective history at this locus. No correlation exists between nucleotide diversity at the R-genes in *Capsella rubella* and the same R-genes in *Arabidopsis thaliana* (Figure 1B; p = 0.487, r² = 0.318), although the shared pattern of high polymorphism in AT1G56540 is evident.

## Discussion

Overall, patterns of polymorphism at disease resistance loci show clear departures from the reference genes. In particular, the difference in synonymous and non-synonymous nucleotide diversity statistics is striking (Table 1). Although *C. rubella* has largely reduced diversity compared to *C. grandiflora* throughout its genome, it has even higher average diversity than *C. grandiflora* at this set of disease resistance loci (Table 1). Similarly, the average synonymous diversity at this set of R genes in *C. rubella* is five times higher than the genome-wide pattern, while in *C. grandiflora* average diversity is in fact slightly lower in this set of genes. This is a strong indication that balancing selection, either ongoing or historical, may have maintained ancestral polymorphism at some R-genes in *C. rubella*, despite a severe population bottleneck and mating system shift. The significant reduction in levels of differentiation between the species, as measured by Fst, as well as higher proportions of shared (AT1G56540) and unique polymorphism in *C. rubella* (Table 3) is also in line with the maintenance of variation due to balancing selection. Although we don't see a significant elevation of synonymous Tajima's D, as expected under some parameter space for balancing selection, the trend is towards elevated Tajima's D values, particularly at the loci showing elevated diversity (Table 1). It is possible that the lack of significance for Tajima’s D reflects a lack of power due to sample size; because our sequencing strategy was matched with our reference genes, sample sizes are relatively low. Although our basic conclusions about the retention of balancing selection should be robust, future genome-wide studies with larger sample sizes and more information on the allele frequency spectrum of flanking loci may also detect a significant skew in the frequency spectrum in these regions.

The impact of selective forces has previously been shown to overcome genome wide polymorphism patterns resulting from demography or mating system. In *Arabidopsis thaliana*, nucleotide diversity was over five times higher at the R-gene RPP13 compared to its close outcrossing relative, *A. lyrata*, despite lower genome-wide diversity in the inbreeder [42].

It is important to consider the extent to which the patterns described above are reflective of individual unusual loci vs. the set of R-genes as a whole. Clearly, a global assessment of the retention of polymorphism in *C. rubella* due to balancing selection will require a genome-wide analysis, but our present data allows us to get a first sense of the variance in selection patterns across R genes. The elevated levels of non-synonymous nucleotide diversity in several of the R-genes are indications of non-neutral evolution (Table 1). This pattern is consistent with balancing selection, as well as relaxed selective constraint on the R-genes. However, balancing selection is expected to cause elevated levels of synonymous polymorphism as well, a pattern that was observed in only one of the R-genes in *C. grandiflora* (AT1G56540) and two in *C. rubella* (AT1G56540, AT1G63730) (Table 1). Furthermore, there is a similar excess of non-synonymous divergence across genes, suggestive of relaxed constraint.

The two genes with elevated synonymous diversity therefore show the strongest evidence for balancing selection, and the highly elevated average diversity in *C. rubella* is in large part driven by AT1G63730 that, surprisingly, shows low diversity in *C. grandiflora*. This could reflect an unsampled divergent haplotype or a loss of balancing selection in *C. grandiflora*. Since we recovered PCR amplicons from all individuals for this locus, biased amplification of one allele seems unlikely. However, inspection of new Illumina resequencing data in *C. grandiflora* indicates that the haplotype occurs in *C. grandiflora*, but it simply remains unsampled in this dataset (data not shown). Interestingly, the locus AT1G56540 also shows evidence of balancing selection in *Arabidopsis thaliana* (Figure 1), while AT1G63730 is a candidate for a partial selective sweep in *A. thaliana*[45]. This suggests that particular loci may remain the target of ongoing diversifying and balancing selection over long evolutionary timescales. A third locus, AT1G64070, shows significantly elevated non-synonymous diversity, as well as a non-significant trend towards higher synonymous diversity in *C. grandiflora*. It is possible that this locus is subject to weak balancing selection, or the region we have sequenced is linked to a region under balancing selection, and the loss of variation at this locus in *C. rubella* could reflect a loss of selected diversity. In the case of the R-genes with elevated non-synonymous divergence, but not diversity (*C. grandiflora*: AT1G17600, AT1G27170; *C. rubella*: AT1G27170, AT1G64070, Table 1) this could be due to positive selection or lower levels of constraint on these genes compared with the rest of the genome. However, McDonald-Kreitman tests [51] were not significant for any of these R-genes (data not shown), so there is no evidence for positive selection, and thus we cannot reject the hypothesis that this simply reflects relaxed selective constraint.

Why do we detect more significant differences at R genes compared to the genome-wide average in *C. rubella* than in the ancestral *C. grandiflora*? One possibility might be that PCR problems meant that we failed to amplify more divergent alleles in *C. grandiflora*. However, this would predict a general reduction in our inferred *C. grandiflora* diversity levels in loci with smaller realized sample sizes, but there is no evidence that this is the case (Table 1). Nevertheless, a divergent haplotype at AT1G63670 did remain unsampled in *C. grandiflora*. Additionally, high levels of linkage disequilibrium in *C. rubella* combined with the genome-wide loss of diversity due to the population bottleneck may exaggerate the signal of balanced polymorphism. In highly outcrossing, equilibrium species, recombination events will limit the signal of balancing selection to a very narrow region surrounding the selected site [52], whereas we expect a more extended elevation of diversity in selfing species, creating a much greater difference between regions under balancing selection and the genomic average. Our results highlight the possible importance of balancing selection in generating strong variance in the retention of diversity in bottlenecked, selfing species.

## Conclusions

Our data are consistent with previous studies that found more polymorphism patterns consistent with balancing selection compared to positive selection in surveys of plant disease resistance genes [20,22,37-39,41]. However, there is also a great deal of evidence for relaxed selective constraint on many of the R-genes compared to reference genes, which was also found in the R-genes in *Arabidopsis thaliana*[45], a pattern which may be reflective of conditional neutrality of the loci under environmental conditions where the functional gene is no longer adaptive [44]. In either case, diversity may be maintained by ongoing selection, or neutrally, following a history of balancing selection, at the disease resistance genes in *C. rubella*. Nevertheless, it is also quite possible that the low power of the McDonald-Kreitman test at individual loci is preventing the detection of positive selection on amino acids. Further studies of genome-wide patterns will help assess the degree to which high amino acid substitution at R-genes is driven by recurrent positive selection, weak negative selection, diversifying selection, or relaxed constraint. Two of the seven R-genes in our sample for which we have data from both species, AT1G56540 and AT1G63730, show patterns of polymorphism that are consistent with either present or past balancing selection acting at these loci. The patterns persisted through the speciation, and diversity reduction in *Capsella rubella*, regardless of whether the selective forces are still active. Therefore we can conclude that historical or ongoing balancing selection may play an important role in the differential retention of polymorphism across the genome.

## Methods

### Samples

Frozen leaf material from 8 accessions of *Capsella grandiflora* and 7 accessions of *C. rubella* was used in this study. Samples of *C. grandiflora* were collected in Greece, and those of *C. rubella* were collected from several countries in Europe (Additional file 1: Table S1). Sampling was conducted to largely match the populations and sample sizes of a survey of genome-wide polymorphism at reference genes [5] (Additional file 2: Table S2).

### Genomic region and primer design

The primers used for PCR (Additional file 3: Table S3) were designed by Bakker [45] to amplify fragments in the leucine-rich-repeat regions of R-genes in Arabidopsis. Those included in the study were the primers that were successful in amplifying LRR regions of R-genes in both *Capsella* species. Protein coding domains of the amplified fragments were confirmed in the *Arabidopsis thaliana* genome using the Basic Local Alignment Search Tool (BLAST) program, BlastX, using the default settings [47,53](Additional file 4: Table S4). The single-copy nature of the R-genes was confirmed by the lack of double peaks in the chromatograms for individuals of *C. rubella*, indicating no ‘heterozygosity’ in this species reflective of the amplification of gene duplicates.

### DNA analysis

DNA was extracted from frozen leaf material using the Dneasy Plant Mini Kit (Qiagen), and amplified by polymerase chain reaction (PCR) in 96-well plates using a Master Cycler thermocycler (Eppendorf). Temperature cycles began with 2 min at 94°C, followed by 35 cycles of the following: 20s at 94°C, 20s at 55°C, and 40s at 72°C. When the cycles were completed the samples were kept at 72°C for 4 min, and then cooled to 4°C, after which they were moved to a −20°C freezer and stored until sequencing. Sanger sequencing of PCR products was performed by the Genome Quebec Innovation Center (Quebec, Canada). Chromatograms were analyzed using Sequencher 4.7 (Gene Codes, Ann Arbor, MI). Heterozygous sites were found by first calling secondary peaks at the 35% threshold, followed by manual inspection of all putative heterozygous positions. Sequence data from both forward and reverse sequence strands were used for confirmation. Homologous regions of the sequences in the closely related genetic model plant *Arabidopsis thaliana* were determined using BLAST [53], and aligned to the sequences using the software GeneDoc [48] in order to obtain an outgroup for estimates of nucleotide divergence.

### Sample size

Sequences with chromatograms of poor quality were excluded, as were those with sample sizes of less than 6 haploid sequences, and those containing fewer than 60 synonymous sites. In total, one sequence fragment from the LRR region of each gene was included for each of the nine R-genes (AT1G12290 [Genbank: JX262585 - JX262607], AT1G17600 [Genbank: JX272820 - JX272829], AT1G27170 [GenBank: JX272799 - JX272819], AG1G56540 [GenBank: JX272830 - JX272841], AT1G63730 [GenBank: JX426710 - JX426732], AT1G63740 [GenBank: JX426695 - JX426709], AT1G64070 [GenBank: JX426733 - JX426747], AT3G50950 [GenBank: JX426748 - JX426770], AT5G17680 [GenBank: JX426771 - JX426793], 0020 Additional file 3: Table S3). Sequences from seven of the genes were included for both species, and two were included only in *C. grandiflora*. The R-gene sample sizes are therefore 9 and 7 for *C. grandiflora* and *C. rubella*, respectively.

### Sequence analysis

Population summary statistics for nucleotide diversity, divergence, and frequency were generated using a version of the perl script Polymorphurama [46] modified by the authors. Diversity statistics included two estimators of the population mutation parameter, pi (π) and Watterson's θ_w_. Pi (π) is defined as the average pair wise number of nucleotide differences per site for a sample of DNA sequences [49,54], and Watterson's θ_w_ summarizes the amount of nucleotide diversity based on the total number of segregating sites, and the sample size, in a group of DNA sequences [55]. The frequency spectrum of polymorphism was measured by Tajima's D statistic, calculated by taking the difference between π and θ_w_[56]. Average pair wise divergence (K) was calculated, using *Arabidopsis thaliana* as an out-group, as the average number of nucleotide substitutions per site between species, with a Jukes and Cantor correction [49]. Direction and degree of selection was qualitatively measured using the neutrality index [57], based on the McDonald Kreitman Test [51]. This statistic measures the degree to which the levels of amino acid variation within species depart from the expectations of neutrality. Differentiation between species was measured using Wright’s Fst Statistic [58,59], which uses the amount of variation in SNP allele frequencies between samples of DNA sequences from different groups to determine the degree to which those groups are genetically dissimilar.

In order to detect non-neutral patterns of evolution, summary statistics for the R-genes were compared to a genome-wide sample of ‘reference’ genes from plants of both species from related plant families of equivalent geographic sampling [5], each containing a minimum of 60 synonymous sites and a sample size of six. In total there were 283 neutral genes that met the above criteria for both species*.* R-gene summary statistics were tested for significance in a two tailed test (p < 0.05). The values of summary statistics for which individual R-genes fell within the 2.5% tails of the genome-wide distribution for the different summary statistics were noted. The R-genes as a group were compared to the neutral genome-wide distribution using permutation tests for the means of the summary statistics. For each permutation the means of the summary statistics were calculated for the samples of R-genes in both species, and compared to an equal number of reference genes resampled from the genome-wide dataset for ten thousand permutations, in order to calculate the proportion of permuted datasets for which the mean value was as or more extreme than the disease resistance loci at the one-tailed 2.5% level. Shared, unique, and fixed nucleotide differences at the R-loci between species were calculated using a perl script written by the authors.

Since the primers for the disease resistance genes used in this study were designed for *Arabidopsis thaliana* by Bakker [45], there is complete overlap with the R-genes sampled here and those analyzed in *A.* thaliana [45]. Therefore we correlated values for the nucleotide diversity statistic π, between both *Capsella* species and *Arabidopsis thaliana*. Figures were produced by the statistical software R 2.13.1 [60].

## Competing interests

The authors declare that there are no competing interests.

## Authors’ contributions

Authors GG and SW were responsible for conception and design of the study. GG and TS performed the data collection. Analyses used in this study were completed by GG. The paper was written by GG and SW. All authors read and approved the final manuscript.

## Supplementary Material

Additional file 1 **Table S1.** Locations of the individuals from which the R-gene sequences were sampled. Click here for file

Additional file 2 **Table S2.** Locations of the individuals from which the genome-wide sequences were sampled. Click here for file

Additional file 3 **Table S3.** Primers used for PCR amplification of R-gene fragments. * Data from *C. grandiflora* only. Click here for file

Additional file 4 **Table S4.** BlastX Coordinates and protein coding domains for the R-gene fragments. * Data from *C. grandiflora* only. Click here for file
